# Tobacco harm reduction in Afghanistan: a recipe for improving smokers’ health

**DOI:** 10.1186/s13011-023-00517-2

**Published:** 2023-01-22

**Authors:** Attaullah Ahmadi, Ali Rahimi, Mohammad Faisal Wardak, Hamidullah Ahmadi, Don Eliseo Lucero-Prisno

**Affiliations:** 1 École des Hautes Études en Santé Publique, Paris, France; 2 Medical Research Center, Kateb University, Kabul, Afghanistan; 3 Medical Faculty, Herat University, Students Street, Herat, 3001 Afghanistan; 4 American University of Afghanistan, Kabul, Afghanistan; 5 Faculty of Management and Development Studies, University of the Philippines Open University, Los Banos, Laguna Philippines

**Keywords:** Tobacco, Harm reduction, Smoking cessation, E-cigarettes, Tobacco harm reduction, Afghanistan

## Abstract

Tobacco Harm Reduction (THR) offers a promising approach to addressing the significant burden of smoking in Afghanistan. Over three million Afghans smoke daily, making it a leading cause of preventable deaths in the country. While the previous Afghan government implemented various tobacco cessation policies and strategies, these measures were only partially effective in reducing the number of smokers or smoking-related deaths. In 2021, community-based initiatives in Kabul and Herat started advocating for Tobacco Harm Reduction (THR) as a novel, realistic, and practical approach proven to promote smoking abstinence and minimize tobacco harm. However, implementing THR strategies in Afghanistan faces numerous challenges, including a lack of governmental support, funding issues, unfavorable market conditions, the high cost-effectiveness of THR products, and misconceptions about these products. To effectively promote THR in Afghanistan and overcome these challenges, it will be necessary to implement THR policies that support THR products for smokers, regulate the market for these products, produce them locally with healthcare professional oversight, conduct more engaging advocacy campaigns, and secure domestic sponsors.

## Background

Tobacco products are used by an estimated 1.3 billion individuals globally, with more than 80% living in low- and middle-income (LMIC) countries [1, 2]. Tobacco use is the leading preventable cause of disease, disability, and death. Smoking tobacco can result in lung cancer, chronic obstructive pulmonary disease (COPD), and cardiovascular diseases [3]. More than 7 million deaths are directly related to tobacco use, while around 1.2 million are related to nonsmokers exposed to second-hand smoke [2].

To address the significant burden of tobacco on public health, the World Health Organization (WHO) adopted the Framework Convention on Tobacco Control (FCTC) in 2003 [4]. The FCTC was the first world public health treaty to promote smoke-free legislation and implement a national tobacco control program based on MPOWER policies. These policies include: 1) monitoring tobacco consumption and the effectiveness of preventive measures; 2) protecting people from tobacco smoking; 3) offering aid to quit tobacco use; 4) warning about the dangers of tobacco; 5) enforcing restrictions on tobacco advertising, promotion, and sponsorship; and 6) raising tobacco taxes [4]. In 2015, the United Nations included an enhanced tobacco control strategy as part of its third global development goal to strengthen the FCTC in all countries [5].

Nicotine, the addictive component of tobacco, has a brief to no cardiovascular effects and poses no risk of respiratory disorders such as chronic obstructive pulmonary disease or cancer [6]. It correlates to the modern smoking cessation strategy known as Tobacco Harm Reduction (THR), which aims to reduce the health risks associated with tobacco use by encouraging the use of alternative nicotine products such as e-cigarettes, snus, etc. [7]. Smokeless Tobacco (ST), Nicotine Replacement Therapy (NRT), and e-cigarettes can reduce or eliminate exposure to carcinogens and other harmful substances while still supplying nicotine [8]. THR has the potential to lead to one of the most prominent public health breakthroughs in history by significantly decreasing the projected one billion cigarette-caused deaths this century [9].

Tobacco smoking is a leading cause of preventable illness and death [10], and smoking cessation is critical to public health [11]. However, quitting smoking/tobacco is challenging, and even approved smoking cessation therapies have low success rates [12, 13]. Some smokers may also be unwilling or unable to quit [3]. It is because nicotine, like opioids, alcohol, and cocaine, is highly addictive and can cause relapse after tobacco abstinence, leading to low success rates in smoking cessation efforts [3]. A 2006 National Institutes of Health (NIH) report found that about 70% of smokers in the United States want to quit, but fewer than 5% succeed in any given year [14]. Providing low-harm alternatives to smokers can result in a lower total population risk compared to adopting abstinence-focused programs [15]. These alternatives include e-cigarettes; modern ST products like nasal snuff, chewable tobacco, and oral snuff; and NRT products such as nicotine gums, patches, nasal spray, inhalers, and lozenges.

ST products, such as nasal snuff and oral ST, carry significantly fewer health risks than smoking cigarettes, with an estimated risk of only about 2% of the risk of smoking, according to Rodu and Cole [16, 17]. Many epidemiological studies and meta-analyses confirm that using ST products like Swedish snuff is associated with minimal risk of myocardial infarction and stroke compared to smoking cigarettes [3, 9, 18, 19]. Systematic reviews indicate that the cancer risk from ST products, such as snuff and chewing tobacco, used in North America and Europe is lower than that from smoking (nearly 1–2%) [3, 20, 21]. Numerous government surveys [22–26] and one clinical trial conducted in the US [27, 28] suggest that many smokers have successfully quit smoking by switching to ST products such as snuff and snus and that these products are effective in helping smokers become smoke-free.

Due to their ST consumption (i.e., Swedish snus), Swedish men have had the lowest rates of smoking-related cancers of the lung, larynx, mouth, and bladder in Europe over the last 50 years, as well as the lowest percentage of male deaths related to smoking of all developed countries [3]. Snus should be considered a lower-risk product compared to cigarettes, based on the findings of a review study that indicated that snus is a viable alternative to smoking tobacco, is acceptable to consumers, and does not serve as a gateway to smoking cigarettes [29]. These conclusions are consistent with those previously reached by the UK Royal College of Physicians [30]. In 2019, the share of daily smokers among people aged 15 and up in Sweden was 6.4%, the lowest in the European Union [31]. The Swedish tobacco experience proves ST to be safer than smoking tobacco. In Afghanistan, Naswar is a popular dipping ST made from tobacco and lime. It typically comes in dry powder, moist powder, or small polythene bag forms, and people place it in the mouth cavity to achieve a euphoric sensation [32]. Local manufacturers, including those who are illiterate, often produce Naswar without following proper health and safety guidelines.

E-cigarettes and vaping products are often used as alternatives to traditional tobacco products for long-term abstinence or as a smoking cessation aid [33]. They are reusable, with the user only needing to replace the nicotine liquid after a certain period [34]. Vapers, or people who use e-cigarettes, often experience less stigma than smokers, making e-cigarettes more appealing for smokers looking to quit [34]. E-cigarette use can mimic the handling and psychological cues of smoking, making it an effective tool for smoking cessation without experiencing withdrawal symptoms [9]. According to a review study, e-cigarettes are 95% less harmful than tobacco smoking [33]. As a result, vaping has become a popular smoking cessation tool and harm reduction approach among the medical and public health bodies of the United Kingdom [35]. However, it is crucial to note that in many countries, including the United Kingdom and Sweden, THR products (e.g., e-cigarettes and snus) are not allowed to be marketed to youth, as they are considered tobacco-naive individuals [31, 35].

NRT is a newer form of ST that has gained increasing attention in recent years as a safer alternative to smoking. The scientific community has consistently evaluated NRT as a safe and effective quitting tool [36]. A study found that NRT increases the chances of quitting smoking by 50 to 70% [37], and another study found that NRT can help reduce the amount of tobacco use among people who are not ready to quit smoking altogether [38]. Many clinical guidelines recommend NRT as a first-line treatment for those seeking pharmaceutical assistance to quit smoking [36].

In 2007, Afghanistan began implementing various laws to regulate tobacco use, including bans on smoking in most indoor workplaces and public places [39]. These laws also prohibited most tobacco advertising and promotion, required health warnings on product packaging, and imposed an excise tax on cigarettes [39]. In 2010, Afghanistan adopted the WHO’s Framework Convention on Tobacco Control (FCTC) [1, 39]. However, in 2018, an amendment to tobacco control in Afghanistan removed the requirements for health warnings on product packaging [39]. According to data from the World Bank, smoking rates in Afghanistan decreased by around 20% between 2010 and 2020, from 29.1 to 23.3% [40]. While this decrease is noteworthy, it may not have been as significant as desired, and further efforts are necessary to continue reducing tobacco use in Afghanistan [41]. According to a report from the WHO, current smoking rates in Afghanistan are 35.2% among men and 2.1% among women [41]. Without improved policies, smoking-related deaths will reach 1.74 million of the 3.5 million smokers alive, with figures expected to continue to escalate year by year [41]. The WHO identified a number of factors that have hindered effective tobacco control in Afghanistan [41], including: a lack of fines and enforcement funds for violating smoke-free legislation; a lack of mechanisms for citizens to file complaints or for investigations to be carried out; a lack of quit lines or other cessation services; a lack of government expenditure on tobacco control; a lack of national anti-tobacco media campaigns; a lack of bans on international television channels, radio stations, magazines, newspapers, billboards, point-of-sale advertising, or internet advertising of smoking; a lack of fines for violations of advertising bans; a lack of requirements for anti-tobacco advertisements to be shown before, during, or after the broadcast of visual entertainment; and other issues such as ongoing political violence, poverty, tobacco industry interference with tobacco control policies (e.g., political lobbying), rising corruption, failing state and political institutions, and vulnerability to the expansion of the corporate tobacco market due to a weak economy.

The FCTC and its MPOWER strategy have helped reduce tobacco use in high-income and European countries [42] and have also had some success in Afghanistan. However, there are still challenges in implementing tobacco control policies in Afghanistan. Using ST, NRT, and e-cigarettes as alternatives to smoking tobacco may help reduce the negative health consequences of smoking in Afghanistan. This article provides an overview of the current state of tobacco harm reduction in Afghanistan and recommends evidence-based strategies for promoting and effectively implementing harm reduction policies, taking into account the specific context and challenges of the country.

## Commentary

The THR initiative was introduced in Afghanistan in 2021 to help Afghan smokers switch to safer nicotine products to reduce tobacco-related harm and increase their chances of quitting smoking. Through awareness campaigns, THR Afghanistan, with the support of Knowledge Action Change, initiated a project in Kabul and Herat to inform the public about the dangers of smoking and methods for reducing harm. This new initiative, called THR Afghanistan, aims to promote safer products and includes a small but dedicated team of staff and volunteer activists. It primarily used social media to reach the public and targeted health workers and the general public in its efforts to involve more people in community advocacy activities for THR [43].

### Challenges

Some e-cigarettes and NRT products may not be cost-effective and may not be suitable as long-term alternatives to cigarettes. Most THR products in Afghanistan, except for Naswar, are imported, and due to import taxes, vaping and NRT products can cost at least twice as much as in neighboring countries [43]. In addition, the Taliban’s caretaker government, which has been in place since the collapse of the republic’s government, has faced severe international sanctions that have damaged the Afghan economy and made imported products such as NRT products, e-cigarettes, and nicotine liquid scarce and unaffordable in the market [44]. These sanctions, which have affected the current caretaker government and excluded its central bank from the international banking system, have caused extreme poverty and a humanitarian crisis in the country. These sanctions only allow for humanitarian funding, which makes it difficult for THR advocacy and research to receive international funding. Accordingly, the THR initiative lacks proper government support due to the incompetence of the caretaker government. Incompetency and financial vulnerability have previously prevented the government from effectively implementing developmental programs. For example, despite the government’s declaration of a ban on opium poppy cultivation in April 2022, the lack of proper implementation resulted in a threefold increase in the income of the opium industry in Afghanistan [45].

Afghanistan’s geography, with many of its people living in remote areas with no access to media, makes reaching out to them difficult. Due to this lack of access to information about alternative THR products, Afghans are only familiar with Naswar. Even if presented with this information, they may resist scientific reasoning due to their lack of proper education. Additionally, the local and tribal leaders in Afghanistan play a significant role in shaping people’s beliefs and behaviors, and individuals may be more likely to trust and follow the guidance of their leaders than outsider sources or unfamiliar concepts. Therefore, given the challenges outlined above, engaging more people would be a staggering challenge.

Misconceptions and ignorance about THR products and tobacco consumption can present a significant challenge to THR in Afghanistan. Some believe that smokeless tobacco is a gateway to smoking initiation, but it may also be a pathway to smoking cessation, according to some studies [25, 29, 46]. However, other studies have produced inconsistent results, and more research is needed [47]. Many people frequently overestimate the dangers of ST and other THR products, such as NRT and e-cigarettes, and are unaware that these products are less harmful than cigarettes [48, 49]. A UK survey found that many smokers and ex-smokers greatly overestimated the role of nicotine in the primary health harms of smoking and the relative harms of e-cigarettes and NRT compared to smoking [48]. Likewise, a study in Norway found that incorrect beliefs about the risks of ST are common in areas where snus is prevalent. Many people continue to believe that the risk of using snus daily is nearly as high as the risk of smoking, despite estimates from medical experts indicating otherwise [49]. Unregulated and non-hygienic local production of Naswar in Afghanistan has stigmatized ST and led people to overestimate its harm. There is also a concern that youth may become addicted to THR products, which could compromise tobacco prevention efforts and the goal of eliminating all tobacco use.

### Recommendations for the adoption of THR in Afghanistan

To increase the adoption of THR in Afghanistan, we must reevaluate traditional tobacco control policies. This model outlines practical steps for implementing THR in Afghanistan, considering the country’s unique social, cultural, economic, healthcare, regulatory, and taxation landscapes. It also provides examples of successful THR policies from other countries and offers solutions to the main challenges to THR adoption in Afghanistan. By considering the specific characteristics of Afghanistan and drawing on successful THR policies from other countries, we can effectively increase the adoption of THR and improve public health outcomes.

1. Reconsideration of taxing legislation: Reducing import taxes on e-cigarettes and NRT can make these products more affordable and viable alternatives to cigarettes. Health-risk-based tax policies should be established for tobacco products, as some, like ST, pose fewer health risks than cigarettes and should be taxed accordingly. The United States National Bureau of Economic Research found that increasing taxes on low-risk products may discourage smokers from switching to them. According to this study, taxing e-cigarettes at the same rate as tobacco would increase the baseline smoking rate in the US by 8.1% and decrease the quit rate by 25% [50]. The THR initiative could work towards implementing appropriate changes in tax legislation through awareness campaigns and regular meetings with the Ministry of Public Health and other government and health stakeholders. The current political situation in Afghanistan has caused THR to have a long way to go before it can be widely adopted.
2. Securing domestic sponsors: It may be difficult to find financial support for THR advocacy initiatives in Afghanistan due to the interim administration’s lack of focus on these issues and the limitations on foreign funding because of sanctions. Domestic sponsors and large commercial businesses with shared interests in THR may be potential funding sources. Other potential funders include philanthropy, governmental grants, and private company sponsorships. Universities and academic institutions may also provide limited financial support for advocacy research, but this can be hard to obtain. Political reforms that address sanctions may also be necessary to secure funding for THR initiatives in Afghanistan.
3. Collaborations with local and national institutions: Collaboration among pro-THR institutions and organizations is necessary to engage more people in advocacy programs. This strategy has been successful in African countries [51, 52]. NGOs, such as medical associations, labor unions, cultural institutions, local community hubs, and other organizations, can help the advocacy campaign expand and reach more people. These organizations often have staff members throughout Afghanistan, even in hard-to-reach communities, and they are familiar with the local culture and situation. They only need to be trained by THR initiative activists. In a tribal country like Afghanistan, it is also effective to involve local and tribal leaders in raising awareness. Workplaces are also good places to raise awareness, as people may be more likely to trust THR if they see that their community and credible organizations support it. However, the media is also invaluable for informing the public and clearing misunderstandings. In addition to raising awareness, these partnerships can make cost-effective campaigning possible, as volunteer activists often conduct most of the advocacy.
4. Implementing risk information market approach: One way to encourage informed decision-making about tobacco products is by implementing a market approach that includes risk information on all products. It could involve regulating the labeling and marketing of tobacco products based on their relative risks and harmfulness. By including these health warning labels on cigarette and ST packages, such as “Warning: Smokeless tobacco use has risks, but cigarette smoking is far more dangerous”; “Quitting tobacco is ideal, but switching from cigarettes to smokeless tobacco can reduce the health risks to smokers and those around them”; or “Notice: Nicotine does not cause cancer, heart disease, or emphysema” on NRT products, smokers can make informed decisions about the safety of NRT, ST, and e-cigarettes compared to combustible tobacco [3]. Government and health authorities should provide information about the relative health risks of each tobacco and nicotine product rather than implying that all tobacco and nicotine products are equally harmful. To appeal to inveterate smokers and those who have struggled to quit, safer alternatives such as ST and nicotine medications should be promoted and marketed attractively by health authorities [53]. The THR initiative should collaborate with the Ministry of Public Health and other stakeholders to achieve these goals.
5. Promoting domestic THR products: Swedish Snus, a popular product among Swedish people and accepted by the government, is an example of a significantly successful domestic THR product [3, 29–31]. Naswar, a domestic product of Afghanistan, is very similar to Swedish snus and has the same potential to repeat the same THR success in Afghanistan. It only needs to be produced using established policies and procedures at certified pharmaceutical companies. Therefore, the domestic tobacco industry can become part of the solution to cigarette smoking by investing in ST products that meet the required quality standards and the needs of smokers. These companies must follow rules that ensure the quality and safety of their products and allow for proper oversight of their advertisements directed toward smokers. Afghanistan has renowned pharmaceutical and tobacco companies that may leverage numerous tobacco plants to generate enough standard ST products for an effective THR. Domestic production, with government support, would balance the market, favoring ST products and even solving the scarcity of the other THR products. Therefore, the THR initiative will prioritize promoting Naswar, as it is a viable, affordable, locally available, and culturally accepted THR product in Afghanistan. It only needs to be de-stigmatized against combustible cigarettes, as most Afghans believe cigarettes are safer and classier.

The THR Afghanistan program aims to provide information about safer alternatives to tobacco products to adults over 25 years old [43]. While quitting smoking altogether is still the best option, harm-reduction products such as e-cigarettes and snus can help individuals quit. Tobacco-naïve individuals, such as youth, are not intended to be targeted by these products. Youth smoking is a significant issue in Afghanistan, and it may be controversial to include youth in harm reduction initiatives. However, it is crucial to recognize that many young people smoke cigarettes, and excluding them from harm-reduction efforts may not be the most effective way to address the issue. In the initial stages of the THR program, we will focus on providing information to policymakers and other decision-makers rather than directly targeting youth. As more research becomes available, the government should consider the potential role of harm reduction initiatives in addressing youth smoking in Afghanistan. The ultimate decision on harm reduction strategies to address youth smoking in Afghanistan will be left to the government to determine based on the available evidence and best practices.

## Conclusions

The tobacco control measures implemented in Afghanistan have not been consistently effective in significantly reducing the morbidity and mortality related to smoking. However, the THR program offers a promising opportunity to help with smoking cessation and reduce harm from tobacco use in Afghanistan. Implementing THR policies, such as enclosing health warning labels about the relative risks of different tobacco products on the cigarette, ST, and NRT packaging can help smokers make informed decisions and switch to less harmful alternatives as part of offering them an alternative. Health officials should allow the space to promote safer alternatives, like ST and nicotine medications. Because some tobacco products have fewer health risks than cigarettes, they should be priced and taxed based on their relative dangers. A shortage and high cost of e-cigarettes and vaping products, and the abundance of non-standard ST products in the Afghan market, present a significant barrier for smokers looking to switch to non-tobacco or less harmful tobacco products. The government can lower the import tax on e-cigarettes and NRT products and regulate the ST industry to ensure the availability of standard ST products to address the issue of the high cost and shortage of these products in the Afghan market. Naswar is a viable, affordable, locally available, and culturally accepted THR product, making it the most promising option. To increase awareness and understanding of THR, the THR initiative should seek out local and domestic sponsors and collaborate with trusted organizations, community leaders, and the media. Despite the challenges faced by THR in Afghanistan since the collapse of the government in 2021, this initiative can utilize specific interventions and opportunities to minimize these challenges.

## Data Availability

Not applicable.

## Abbreviations

ST: Smokeless Tobacco
NRT: Nicotine Replacement Therapy
FCTC: Framework Convention on Tobacco Control
THR: Tobacco Harm Reduction
WHO: World Health Organization
LMIC: Lower- and Middle-Income Countries
NGO: Non-Governmental Organization
COPD: Chronic Obstructive Pulmonary Disease
